# Diagnostic performance and clinical implications for enhancing a hybrid quantitative flow ratio–FFR revascularization decision-making strategy

**DOI:** 10.1038/s41598-021-85933-9

**Published:** 2021-03-19

**Authors:** Joyce Peper, Robbert W. van Hamersvelt, Benno J. W. M. Rensing, Jan-Peter van Kuijk, Michiel Voskuil, Jurriën M. ten Berg, Jeroen Schaap, Johannes C. Kelder, Diederick E. Grobbee, Tim Leiner, Martin J. Swaans

**Affiliations:** 1 Department of Cardiology, St. Antonius Hospital, Koekoekslaan 1, 3435 CM Nieuwegein, The Netherlands; 2 Department of Radiology, University Medical Centre Utrecht, Utrecht, The Netherlands; 3 Department of Cardiology, University Medical Centre Utrecht, Utrecht, The Netherlands; 4 Department of Cardiology, Amphia Hospital, Breda, The Netherlands; 5 Julius Centre for Health Sciences and Primary Care, University Medical Centre Utrecht, Utrecht, The Netherlands

**Keywords:** Cardiology, Interventional cardiology, Coronary artery disease and stable angina

## Abstract

Invasive fractional flow reserve (FFR) adoption remains low mainly due to procedural and operator related factors as well as costs. Alternatively, quantitative flow ratio (QFR) achieves a high accuracy mainly outside the intermediate zone without the need for hyperaemia and wire-use. We aimed to determine the diagnostic performance of QFR and to evaluate a QFR–FFR hybrid strategy in which FFR is measured only in the intermediate zone. This retrospective study included 289 consecutive patients who underwent invasive coronary angiography and FFR. QFR was calculated for all vessels in which FFR was measured. The QFR–FFR hybrid approach was modelled using the intermediate zone of 0.77–0.87 in which FFR-measurements are recommended. The sensitivity, specificity, and accuracy on a per vessel-based analysis were 84.6%, 86.3% and 85.6% for QFR and 88.0%, 92.9% and 90.3% for the QFR–FFR hybrid approach. The diagnostic accuracy of QFR–FFR hybrid strategy with invasive FFR measurement was 93.4% and resulted in a 56.7% reduction in the need for FFR. QFR has a good correlation and agreement with invasive FFR. A hybrid QFR–FFR approach could extend the use of QFR and reduces the proportion of invasive FFR-measurements needed while improving accuracy.

## Introduction

The most frequently used reference test for the assessment of significant obstructive coronary artery disease (CAD) is invasive coronary angiography (ICA)^1,2^. However, the correlation between anatomically significant stenosis and physiological reduction of myocardial blood flow is weak^3^. Fractional flow reserve (FFR), a physiological test that serves as a proxy for myocardial blood flow, can be used in addition to ICA to assess the hemodynamical impact of a stenosis. Application of FFR has been proven cost-effective for the management of patients suffering from CAD compared to ICA-guided therapy by improving clinical outcomes and reducing stent implantations and thereby costs^4–6^. Nevertheless, it is used in less than 10% of patients due to a combination of procedural and operator related factors as well as high costs^7^.

Recent developments in functional coronary imaging may overcome this problem by allowing for wire- and hyperaemia-free FFR measurements for the assessment of physiological significant stenosis. Quantitative flow ratio (QFR) is a recently developed coronary-based physiological test to calculate a virtual FFR value. QFR is computed by applying (simplified) computational fluid dynamics principles on a three-dimensional model of the coronary artery constructed from two invasive angiographic views^8–11^. Flow-velocity information acquired by Thrombolysis in Myocardial Infarction (TIMI) frame counting can be added to improve the calculations. QFR was recently evaluated in a meta-analysis and was shown to have a sensitivity of 0.89 (95% CI 0.83–0.95) and a specificity of 0.89 (95% CI 0.87–0.92) to diagnose functionally significant CAD (FFR ≤ 0.80)^12^.

Since there are no results of (randomized) outcome studies available, a QFR-only strategy may perhaps not be feasible and safe yet. Furthermore, imperfect correlation between FFR and QFR might result in different treatment decisions based on QFR and FFR measurements, especially when close to the QFR cut-off value of 0.80 (0.75–0.85), also known as the intermediate zone. The low adoption of functional lesion assessment by invasive FFR combined with the high classification agreement between QFR and FFR outside of the intermediate zone provide an opportunity for a combined QFR–FFR strategy. A hybrid QFR–FFR strategy, in which only lesions with intermediate QFR values would require FFR measurements, may seem the most suitable clinical application of QFR. Various QFR-hybrid limits for defining the intermediate zone have been proposed based on 90–95% sensitivity–specificity, namely QFR-treat limits between 0.75 and 0.78 and QFR-defer limits between 0.85 and 0.87^8,10,13,14^. However, these QFR–FFR hybrid limits have only been validated in the cohorts from which they were derived. To our knowledge, this is the first study that assesses the diagnostic value of a QFR–FFR hybrid strategy in an independent, real-world cohort.

Accordingly, the aim of this study was to determine (1) the diagnostic performance of QFR compared to FFR and (2) to evaluate the impact of QFR and QFR–FFR hybrid strategies on the proportion of avoided invasive FFR measurements in consecutive enrolled patients whilst matching the diagnosis of a FFR strategy.

## Methods

### Patient population

The present study is a multicentre study of patients who underwent CT, ICA and FFR to compare QFR versus FFR. All consecutive patients undergoing these diagnostic tests for diagnosing CAD between October 2009 and October 2017 at the St. Antonius Hospital Nieuwegein and all consecutive patients between June 2012 and July 2016 at the University Medical Centre Utrecht were retrospectively enrolled. Three-dimensional quantitative coronary angiography (3D-QCA) and QFR were performed and compared with the reference standard, invasive FFR. 517 vessels in 378 patients were screened. Patients with (1) lack of 2 optimal angiographic views; (2) overlap of other vessels with the lesion or areas around the lesion in the vessel of interest; (3) foreshortening of the target coronary artery in one or both angiographic acquisitions; (4) insufficient contrast injection; (5) location of the lesion of interest at the ostial left main coronary artery or ostial right coronary artery; (6) prior percutaneous coronary intervention (PCI) of vessel of interest (7) bypass grafts were excluded. Since this concerned a retrospective study, the Medical Ethics committees of both institutions (Medical Research Ethics Committees United [MEC-U] and Medisch Ethische Toetsingscommissie Utrecht [METC Utrecht]) and both local institutional boards approved the protocol and waived the need for informed consent. The ethical principles for medical research on human beings of the Declaration of Helsinki and the Good Clinical Practice Guidelines were followed.

### ICA/FFR

Invasive coronary angiography was performed according to the standardized protocol used in both hospitals. ICA biplane views were acquired via either femoral or radial artery access. The coronary artery tree was fully examined for presence of stenosis. Invasive FFR measurements were acquired for clinical indications unrelated to this study using a pressure wire passed beyond the stenosis. Patients suffering from visually assessed intermediate stenosis, defined as a diameter reduction between 50 and 90%, or multivessel disease were subsequently assessed by measuring FFR to determine its functional severity^15^. The pressure gradient across the stenosis was measured during an intracoronary adenosine bolus or continuous intravenous infusion of adenosine at 140 µg/kg/min. The exact location of the wire during measurement was recorded. Vessel-based analysis was performed from which diagnostic accuracy of QFR was determined. To compare and assess the diagnostic performance of QFR, the clinical standard of FFR ≤ 0.80 indicating hemodynamically significant stenosis was applied.

### QFR

3D-QCA analysis and the computation of QFR were done using dedicated QAngio software (QAngio XA 3D 1.1, Medis Medical Imaging System, Leiden, The Netherlands) by a QFR-certified observer while blinded for the FFR results and the treatment decision. The analysis was performed as previously described^11^. In brief, two angiographic views, at least 25° apart, were selected based on the least foreshortening of the stenosis and a minimum overlap between the main vessels and side branches. In both views, the end-diastolic frames guided by ECG (if available) were used for analysis and an anatomical landmark was indicated as reference point. The most proximal and distal points were indicated, and vessel contours were automatically extracted. Manual corrections were made if needed. A 3D reconstruction of the vessel was made for the 3D-QCA and minimum lumen diameter, reference vessel diameter, percentage diameter stenosis and lesion length were extracted. QFR calculations were performed on the anatomical information acquired by 3D-QCA in combination with modelled hyperaemic flow velocity with as input TIMI frame count analysis^16^.

### QFR–FFR hybrid approach

**Figure 1:**
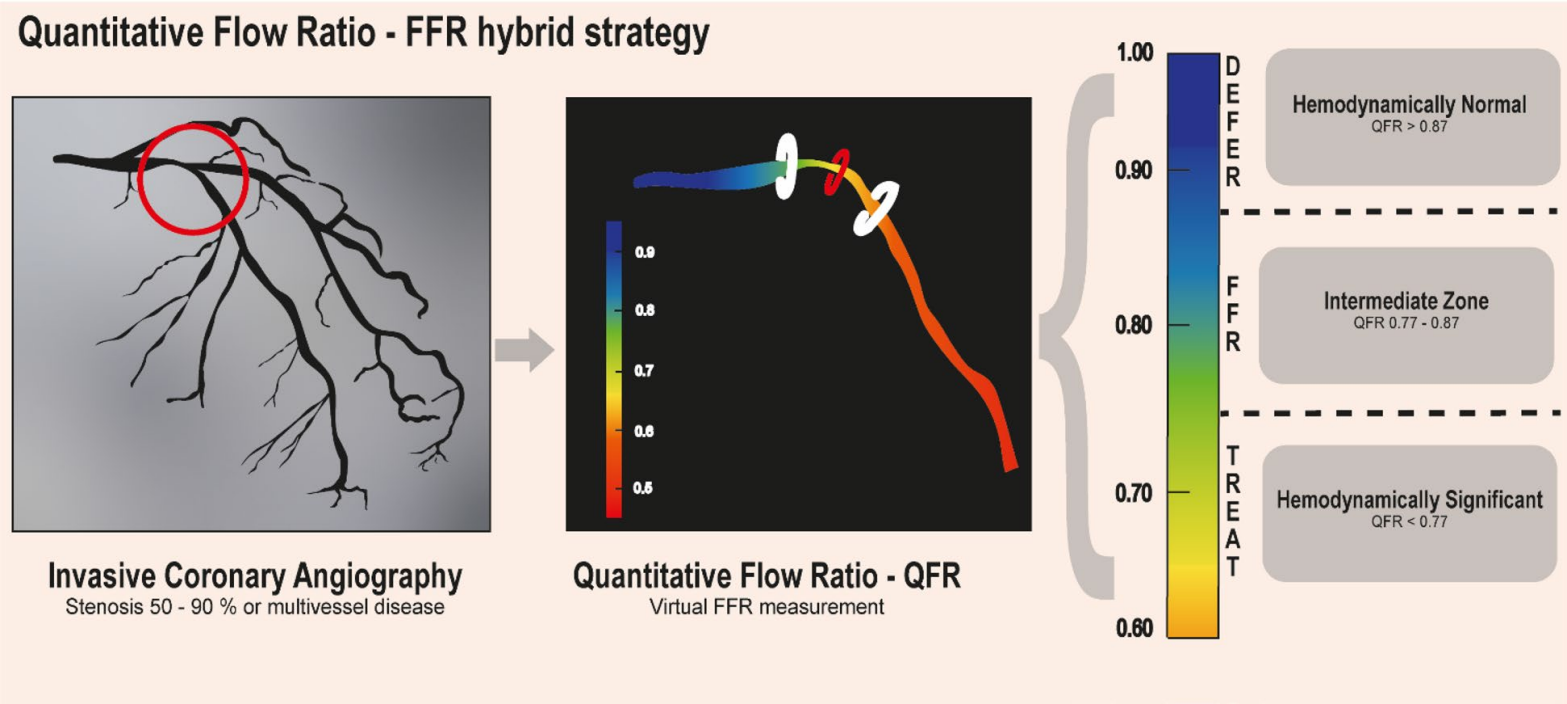
Study overview QFR – FFR hybrid strategy. All 50–90% stenosis or multivessel disease on invasive coronary angiography are assessed by QFR using dedicated software (QAngio XA 3D 1.1, Medis Medical Imaging System, Leiden, The Netherlands). Based on the QFR-value, lesions are categorized into hemodynamically normal (QFR > 0.87), intermediate zone (QFR between 0.77 and 0.87) or hemodynamically significant (QFR < 0.77). Only lesions in the intermediate zone are further assessed by FFR. In our population, this leads to a reduction of invasive FFR measurements of 56.7%. *FFR* fractional flow reserve, *QFR* quantitative flow ratio.

The hybrid approach as proposed in this study includes an intermediate zone of QFR values around the cut-off point to define hemodynamically significant CAD for which FFR measurements need to be performed (Fig. 1). The QFR–FFR hybrid approach was modelled using the limits as proposed in the FAVOR II Europe-Japan study^8^. Normal QFR was defined as QFR > 0.87 (hemodynamically normal), abnormal QFR was defined as QFR < 0.77 (hemodynamically significant) and the intermediate zone was defined as QFR 0.77–0.87 (non-conclusive). In addition, multiple limits (per 0.01 QFR-units) for normal (QFR-defer) and abnormal (QFR-treat) were simulated. The proportions of potential adenosine- and wire-free (FFR-free) procedures defined as the fraction of stenosis outside the intermediate zone, were calculated.

### Statistical analyses

To compare and assess the diagnostic performance of both QFR and the QFR–FFR hybrid approach, the clinical standard of FFR ≤ 0.80 indicating hemodynamically significant stenosis was applied. The same threshold of ≤ 0.80 was applied for the QFR measurements. For the statistical analyses, the distribution of continuous variables was assessed using histograms and Q–Q plots. Continuous variables were expressed as means and standard deviations (SD) if normally distributed or medians and 25–75% interquartile ranges (IQR) if non-normally distributed. Categorical variables were represented as totals and percentages. Diagnostic performance on a per vessel-basis as sensitivity, specificity, positive predictive value (PPV), negative predictive value (NPV) and accuracy were calculated. The variables were calculated as proportions with a 95% confidence interval. The correlation, differences, and diagnostic performance between QFR and wire-based FFR were further assessed using Pearson correlation coefficient, Bland–Altman plots, receiver operating characteristics-curves (ROC-curves) and area under the ROC curve (AUROC-curve). QFR–FFR hybrid approaches using various cut-off points for the intermediate zone in which FFR is measured were simulated. All statistical analyses were performed using R statistical software (www.r-project.org, version 3.6.2).

## Results

**Figure 2:**
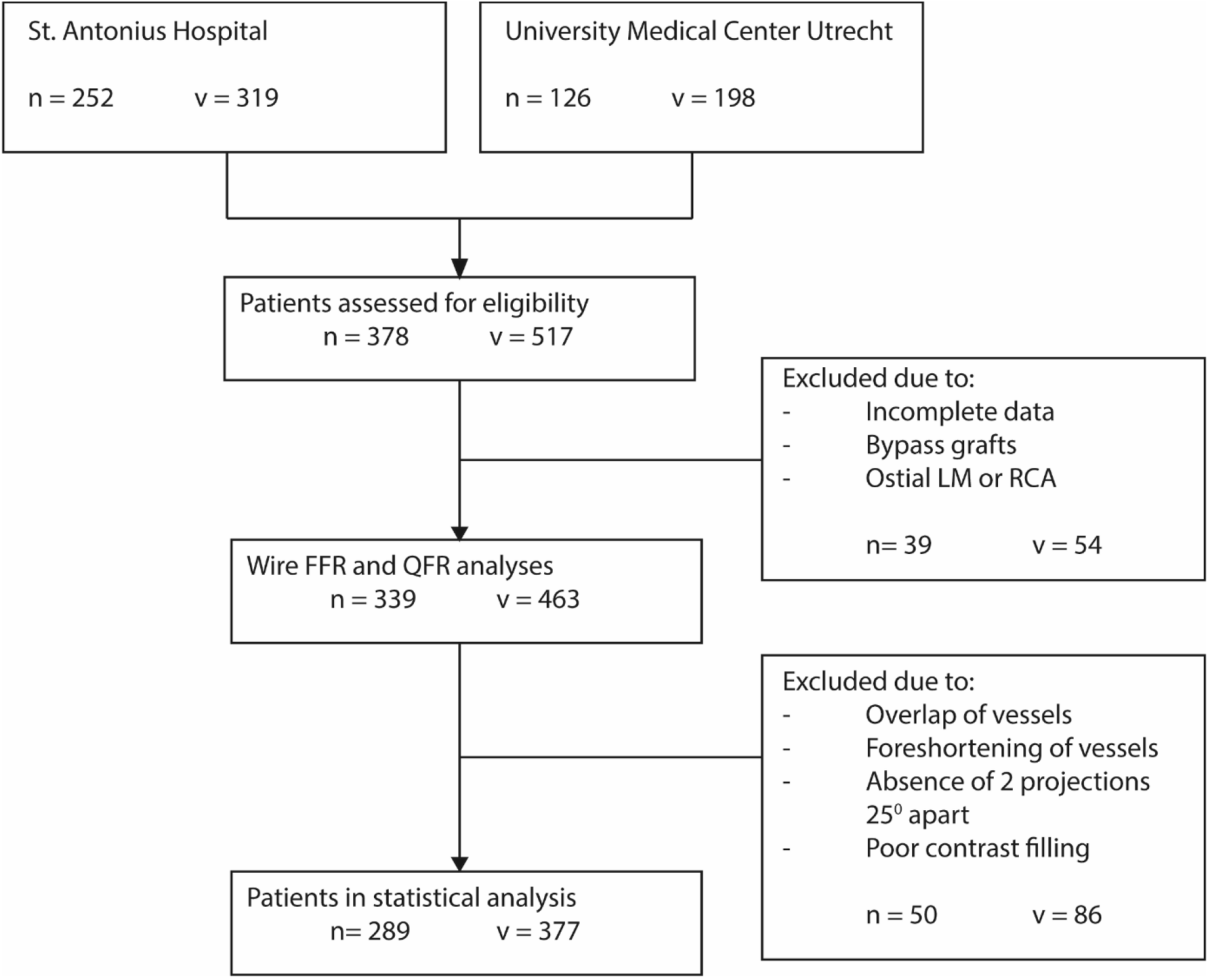
Study enrolment flow chart. *FFR* fractional flow reserve, *LM* left main coronary artery, *N* number of patients, *QFR* quantitative flow ratio, *RCA* right coronary artery, *V* number of vessels.

A total of 378 (252 patients of St. Antonius Hospital Nieuwegein and 126 patients of University Medical Centre Utrecht) patients were identified for potential inclusion in this study. Patients with a history of coronary bypass grafting or lesions in the ostia of the left main or right coronary artery (RCA) (n = 39) were excluded. 339 patients were evaluated by QFR and due to overlap, foreshortening of the stenosis, lack of contrast or lack of two angiographic projections at least 25 degrees apart, an additional 50 patients were excluded. Therefore, 289 patients and 381 vessels were included in the statistical analyses (Fig. 2). Patient characteristics and the characteristics of the analysed vessels are presented in Table 1. Approximately 73.0% of the study cohort was male, the mean age was 64.1 ± 10.4 years and 51.9% had at least three cardiovascular risk factors. On average, the FFR was 0.81 and 169 vessels (44.4%) had a significant stenosis, while the mean QFR was 0.82 and 172 (45.1%) had a significant stenosis.

**Table 1:** Baseline and procedural characteristics.

**Baseline characteristics**	
N (patient)	289
Male (%)	211 (73.0)
Age (years)	64.1 (10.4)
BMI (kg/m^2^)	27.4 (4.1)
Previous PCI (%)	45 (15.6)
eGFR (ml/min/1.73 m^2^) (median (IQR))	86.1 (70.1–96.7)
**Risk factors**	
Hypercholesterolemia (%)	196 (67.8)
Smoking (current or past) (%)	136 (47.1)
Diabetes (%)	53 (18.3)
Family history of CAD (%)	151 (53.0)
Hypertension (%)	213 (73.7)
Risk factors ≥ 3	150 (51.9)
**Procedural characteristics**	
N (vessel)	381
Lesions (%)	
RCA	195 (51.2)
Left main stem	7 (1.8)
LAD	117 (30.7)
LCx	35 (9.2)
Diagonal branch	13 (3.4)
Other	14 (3.7)
Access site a. radialis (%)	238 (62.5)
Contrast (ml) [median (IQR)]	115 (85.0–130.0)
Diameter stenosis (%)	48.5 (9.4)
Lesion length (mm)	20.3 (12.7–24.1)
Minimal lumen diameter (mm)	1.35 (0.38)
Reference diameter (mm)	2.63 (0.57)
Area stenosis (%) [median (IQR)]	62.05 (52.92–71.05)
FFR	0.81 (0.08)
FFR ≤ 0.80 (%)	169 (44.4)
FFR 0.75–0.85	176 (46.2)
QFR	0.82 (0.09)
QFR ≤ 0.80 (%)	172(45.1)
QFR 0.75–0.85	177 (46.5)

Variables are reported as means (standard deviation), unless otherwise specified.

*BMI* body mass index, *CAD* coronary artery disease, *eGFR* estimated glomerular filtration rate, *FFR* fractional flow reserve, *IQR* inter quartile range, *LAD* left anterior descending, *LCx* left circumflex artery, *OM* branch; obtuse marginal branch, *PCI* percutaneous coronary intervention, *QFR* quantitative flow ratio, *RCA* right coronary artery.

### Diagnostic performance

**Figure 3:**
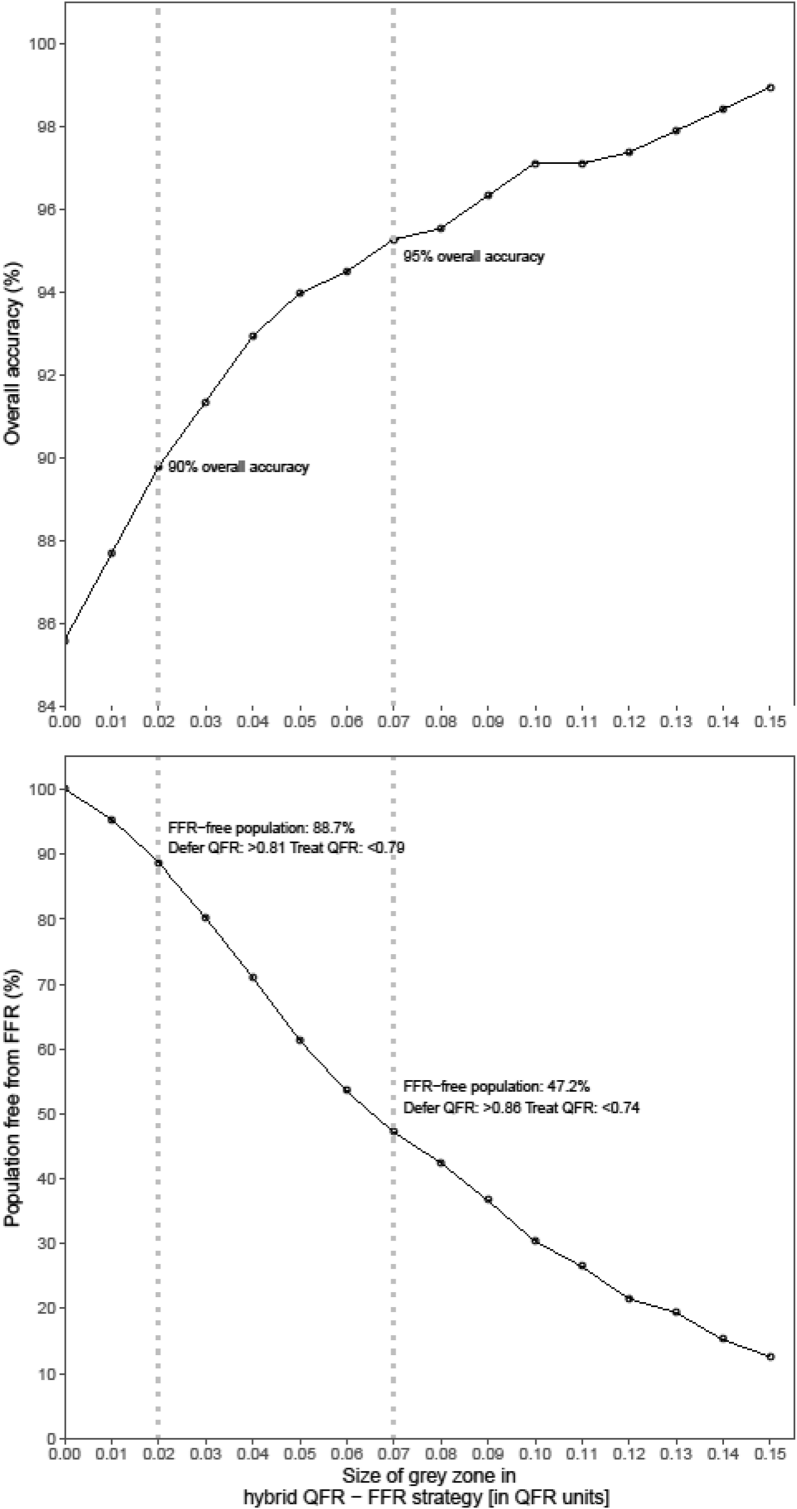
The per-vessel correlation and agreement between QFR and FFR. (**A**) The per-vessel scatterplot shows a good correlation between QFR and FFR (r = 0.72, p < 0.001). (**B**) The Bland–Altman plot shows a small bias of 0.007 ± 0.058 of QFR versus FFR. (**C**) Per-vessels agreement by plotting agreement against the average of invasive FFR and QFR value. QFR shows a high accuracy except for FFR-values around the cut-off 0.80 (0.75–0.85). (**D**) The per-vessels receiver operating characteristic curves (ROC-curves) for QFR and QFR–FFR hybrid approach. The area under the curve was higher for QFR–FFR hybrid approach (AUC = 0.92) versus QFR (AUC = 0.89).

Sensitivity, specificity, NPV, PPV and the accuracy of QFR were 84.6%, 86.3%, 87.6%, 83.1% and 85.6%, whereas the diagnostic test results for the QFR–FFR hybrid approach improved to 88.0%, 92.9%, 86.8%, 93.6% and 90.3%, respectively (Table 2). A scatterplot of QFR and FFR showed a good correlation of 0.72 (p < 0.001). The Bland–Altman plot showed a small bias of 0.007 ± 0.058 of QFR. The area under the ROC curve of QFR was 0.89 and improved to 0.92 with the QFR–FFR hybrid approach. The accuracy plot showed a high accuracy for QFR analysis except for FFR-values around the cut-off 0.80 (Fig. 3).

**Table 2:** Diagnostic test results quantitative flow ratio (QFR).

	_% Diameter stenosis_			_QFR ≤ 0.80_			_QFR–FFR Hybrid approach (QFR: 0.77–0.87)_		
	_Estimate_	_95% CI_		_Estimate_	_95% CI_		_Estimate_	_95% CI_	
_True positive_	_101_			_154_			_103_		
_False positive_	_57_			_10_			_7_		
_False negative_	_68_			_15_			_14_		
_True negative_	_155_			_202_			_92_		
_Sensitivity_	_59.8_	_52.0_	_67.2_	_84.6_	_78.4_	_89.3_	_88.0_	_80.9_	_92.7_
_Specificity_	_73.1_	_66.6_	_79.0_	_86.3_	_81.0_	_90.3_	_92.9_	_86.1_	_96.5_
_NPV_	_69.5_	_65.1_	_73.6_	_87.6_	_82.4_	_91.4_	_86.8_	_79.0_	_92.0_
_PPV_	_63.9_	_57.9_	_69.6_	_83.1_	_76.8_	_88.0_	_93.6_	_87.4_	_96.9_
_Accuracy_	_67.2_	_62.2_	_71.9_	_85.6_	_81.7_	_88.7_	_90.3_	_85.6_	_93.6_
_AUROC_	_0.72_			_0.89_			_0.92_		

The diagnostic performance of ICA based on diameter stenosis and QFR with fractional flow reserve (FFR) as reference standard. Both, FFR ≤ 0.80, diameter stenosis ≥ 50% QFR ≤ 0.80 and FFR ≤ 0.80—intermediate zone QFR (0.77–0.87) are used as diagnostic cut-off values.

*95% CI* 95% confidence interval, *AUROC* area under the receiver operator characteristic curve, *NPV* negative predictive value, *PPV* positive predictive value.

### QFR–FFR hybrid strategy

**Figure 4:**
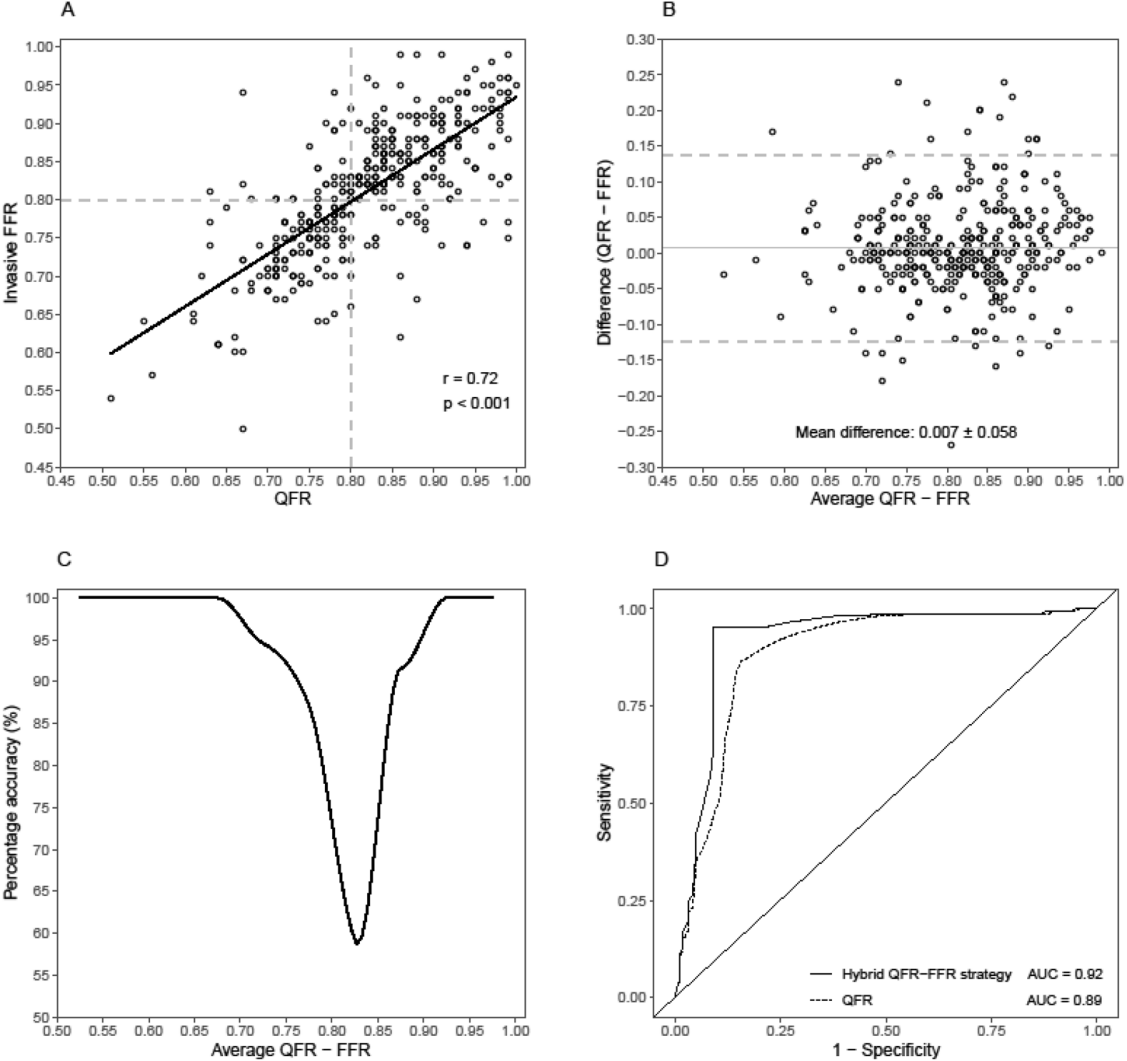
Hybrid revascularization strategy with quantitative flow ratio (QFR) and fractional flow reserve (FFR) reduces the requirement of FFR in clinical practice. (**A**) QFR limits for the intermediate zone on which coronary revascularization decisions can be made without invasive FFR measurement when QFR is < 0.77 (positive predictive value of 93.9%) or when QFR is > 0.87 (negative predictive value of 93.1%). When the QFR-values are between 0.77 and 0.87, invasive FFR is measured and a threshold of 0.80 is applied to guide revascularization. The dots inside the red rectangles represents the false positive and false negative QFR measurements. The dots inside the intermediate zone represent the stenosis that should be classified by FFR. (**B**) A QFR–FFR hybrid approach reduces the number of patients requiring invasive FFR measurements. An accuracy of QFR to FFR of 90% (intermediate zone between 0.79 and 0.83) reduces the invasive FFR requirement by 88.7%.

The diagnostic accuracy of a QFR–FFR hybrid strategy using the pre-specified intermediate zones cut-offs of QFR < 0.77 (positive predictive value of 93.6%) and QFR > 0.87 (negative predictive value of 86.8%), on which coronary revascularization decisions could have been made without invasive FFR measurement was 90.3% (Table 2). The use of the proposed QFR–FFR hybrid strategy would have resulted in a reduction in the use of invasive FFR of 56.7% (Fig. 4). When aiming for an accuracy of QFR of 90% (intermediate zone between 0.79 and 0.83), the proportion of patients in whom invasive FFR could have safely been omitted increased to 88.7%.

## Discussion

In this multicentre study, we determined the diagnostic accuracy of QFR versus FFR. A high diagnostic accuracy of 85.6% was found, while having minimal bias and sufficient discrimination. This study also showed that a QFR–FFR hybrid approach improves the revascularization decision-making strategy over QFR alone. This hybrid approach has the potential to reduce the number of invasive FFR-measurements by more than half, while maintaining a high accuracy-level (accuracy of 90.3%).

### Diagnostic performance

Several studies have been performed to assess the diagnostic performance of QFR. The first prospective study, FAVOR pilot, included 84 vessels in 73 patients. Significant stenoses were found in 27 vessels (32.1%)^11^. A good correlation (r = 0.77, p < 0.001) and agreement between FFR and QFR (mean difference = 0.001, SD 0.059) were found. Diagnostic performance measurements as sensitivity, specificity, PPV, NPV and accuracy on a per vessel basis of 74%, 91%, 80%, 88% and 86% were observed. The subsequent prospective multicentre FAVOR II studies (performed in China, Japan and Europe)^8,9^ also reported good correlations (r = 0.86, p < 0.001 and r = 0.83, p < 0.001 ) and small mean difference between FFR and QFR (− 0.01 SD 0.063 and 0.01 SD 0.06). The sensitivity, specificity, PPV, NPV and accuracy on a per vessel basis were 95%, 92%, 86%, 97% and 93% in the FAVOR II China and 87%, 87%, 76%, 93% and 87% in the European and Japanese cohorts. The WIFI II, a prospective multicentre sub-study of the Dan-NICAD study, included 172 patients and 255 lesions of which 28% of the lesions were functionally significant^10^. A correlation of r = 0.70 (p < 0.001) was observed and precision with a mean difference of 0.01 (SD 0.08). The sensitivity, specificity, PPV, NPV and accuracy on a per vessel basis were 77%, 86%, 75%, 87% and 83%. Our study further corroborates the diagnostic accuracy found in these prior studies.

### QFR–FFR hybrid approach

In the FAVOR II Europe-Japan study, a QFR intermediate zone between 0.77 and 0.87 was proposed, which formed the basis for the present study^8^. FFR assessment could have been avoided in 64% when using the hybrid strategy. Similar results were found in the WIFI II study where an intermediate zone of 0.78–0.87 would have saved pressure wires and adenosine in 68% of the lesions^10^ and by Smit et al. who proposed an intermediate zone with cut-off limits 0.77–0.86 resulting in an accuracy of 93.8% and a FFR reduction of 70.1%^14^. In the current study, we validated the 0.77–0.87 intermediate zone in an independent cohort and found a slightly lower rate of avoided FFR (56.7%), and comparable diagnostic accuracy(93.8% accuracy in this study versus 95% in the FAVOR II study) This might be caused by the difference in proportion FFR-values around the cut-off (0.75–0.85) of 32% in the FAVOR II study versus 46.2% in the present study^8,9^. A second explanation could be found in the different proportion of the measured vessels, whereas the RCA was more often included in the present study (50%) as compared with the FAVOR II study (22%).

### Clinical implications and perspectives

**Figure 5:**
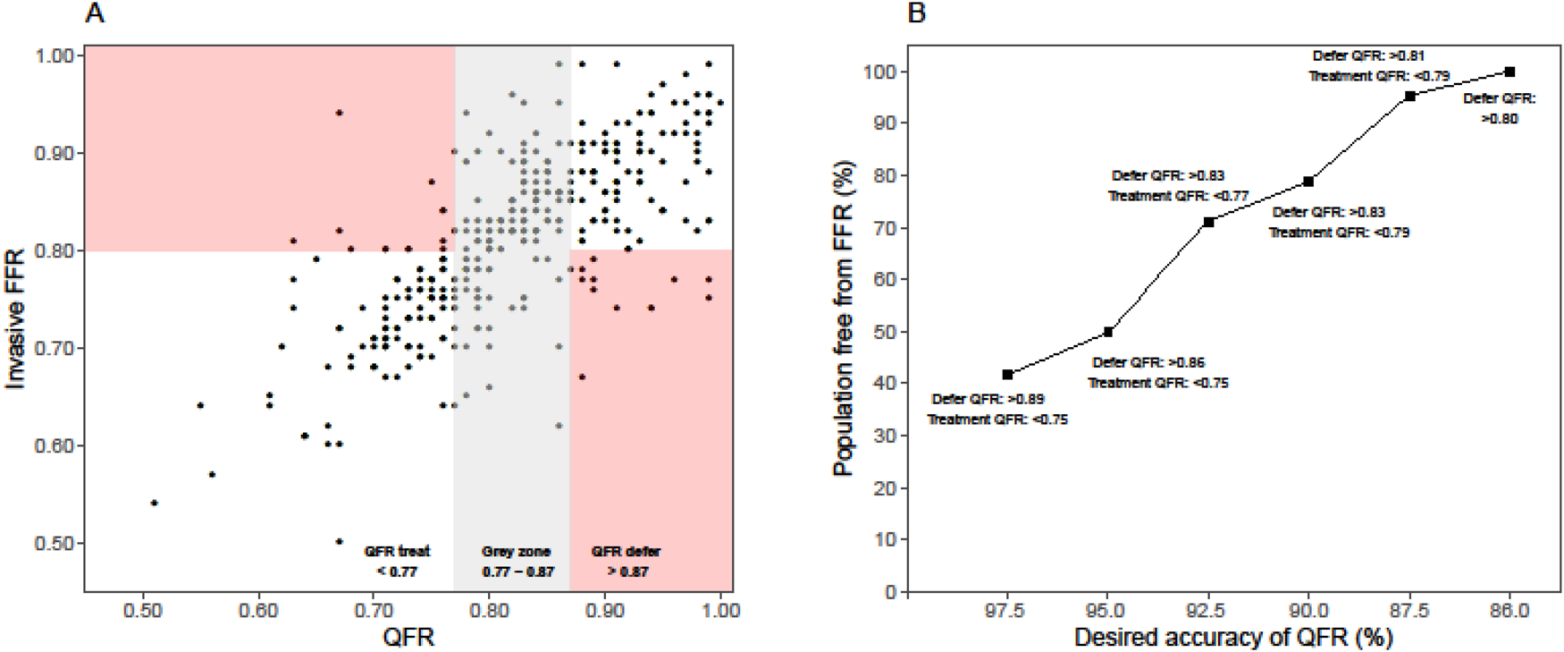
Effect of hybrid QFR–FFR strategies on the population free from FFR and the overall accuracy. Population free from invasive FFR measurements and the overall agreement depends on the size of the intermediate zone: the larger the window of QFR values, the higher the overall accuracy (upper panel). However, this decreases the percentage of patients free from invasive FFR (lower panel).

In this study we showed that the proportion of patients in whom invasive FFR measurements can be avoided and the accuracy both depend on the window of the QFR limits; the wider the intermediate zone, the higher the overall accuracy and the lower the proportion of patients in whom invasive FFR might be safely omitted (Fig. 5). An overall accuracy of 95% using an intermediate zone of 0.07 (0.74–0.86) could reduce the proportion of patient in whom FFR measurements can be omitted to 47.2%. Previously, FFR-guided revascularization has been shown to reduce stent implantations and improve long-term outcome when compared to angiography-guided strategy^4–6^. However, in routine clinical practice FFR is not used very often due to need for hyperaemia induction and a pressure-wire^7^. Hyperaemia might cause patient symptoms and prolongation of the procedural time and the use of a pressure-wire is costly, might lead to procedural complications and can be challenging in tortuous arteries. Other limitations of FFR are waveform artefacts or drift that impacts the accuracy of FFR^17^. Instantaneous wave-free ratio (iFR) overcomes the need of hyperaemia and its negative side effects, but does require invasive measurements.

One of the benefits of QFR is that a virtual FFR can be computed in approximately 5 min (IQR 3.5–6.1) minutes compared to the average of 7 min needed to perform FFR (IQR: 5.0–10.0 min)^8,9^. Moreover, QFR-computation does not require induction of hyperaemia, the hazard of passing an intracoronary wire, or additional equipment, training, or cost for measuring invasive FFR. A QFR–FFR hybrid strategy could potentially increase the adoption of QFR by retaining a high accuracy while decreasing the number of invasive FFR measurements, especially whilst awaiting randomized outcome trials. One downside of the hybrid QFR–FFR approach would be that in addition to the time required to perform FFR, an additional 5 min of QFR time is needed (total time 13 min) in the case of an QFR-value within the intermediate zone.

Another application of QFR is as gatekeeper for hospitals not capable to perform FFR for referrals to hospitals where FFR and PCI can be performed. Currently these hospitals assess the severity of a lesions visually, although visual assessment alone is known to be inaccurate for the assessment of functional significant CAD^14,18^. It has been shown that QFR has a high diagnostic accuracy as well as a high NPV and PPV for the diagnosis of functionally significant CAD. This suggests that QFR could be safely used as gatekeeper. However, randomized studies are needed to confirm the diagnostic value of QFR for this purpose.

## Limitations

This study has several limitations inherent to its retrospective character such as selection bias, which might influence the feasibility and reliability of the QFR analysis. Most limitations are related to imaging quality; a substantial number of patients (14.7%) could not be included in the analysis due to suboptimal projections and/or lack of good quality images. It can be assumed that a prospective study would have less exclusions as the clinician would be more focused on optimal image quality suitable for QFR analysis. Moreover, the QFR analysis could not be applied to ostial lesions and bypass grafts resulting in the exclusion of approximately 10% of the patients and therefore decreasing the applicability of the software. Another limitation could be found in the small differences in vessel characteristics in our study compared to previous studies. Most of target vessels in this study were the right coronary artery, whereas in most of studies reporting on QFR the most common target vessel was the left anterior descending coronary artery. Although our study is performed retrospectively, the vessel characteristics and diagnostic test results are comparable to those observed in prospective studies as the FAVOR II China, Europe-Japan and the WIFI II implicating the limited effect of the differences in vessel characteristics. More importantly, the patients and FFR-measurement included in this study were not specifically performed for study purposes and therefore reflect the clinical practice and the potential benefits of physiological FFR-measurements in addition to visual assessment.

## Conclusion

In conclusion, QFR has a good correlation and agreement with invasive FFR and a high diagnostic performance. With a QFR–FFR hybrid approach, QFR can effectively reduce the number of invasive FFR-measurements with high PPV and NPV. In this way, FFR can be used for equivocal cases only. A hybrid method could potentially increase the adoption of QFR and expand the utilization of physiology-guided decision making in clinical practice while awaiting the results of clinical outcome studies.

## Data Availability

The (anonymized) clinical data, methods used in the analysis, and materials used to conduct the research can be requested by qualified researchers who engage in independent scientific research, and could be provided following review and approval of a research proposal. Data requests can be submitted at any time by contacting the corresponding author.

## Abbreviations

3D-QCA: Three dimensional quantitative coronary angiography
AUROC-curve: Area under the receiver operating characteristic-curve
CAD: Coronary artery disease
CT: Computed tomography
ECG: Electrocardiography
FFR: Fractional flow reserve
ICA: Invasive coronary angiography
iFR: Instantaneous wave-free ratio
IQR: Inter quartile range
LAD: Left anterior descending artery
LCx: Left circumflex artery
LM: Left main
NPV: Negative predictive value
PCI: Percutaneous coronary intervention
PPV: Positive predictive value
QFR: Quantitative flow ratio
RCA: Right coronary artery
ROC-curve: Receiver operating characteristics curve
SD: Standard deviation
TIMI: Thrombolysis in myocardial infarction

## References

[1] Force T (2018). 2013 ESC guidelines on the management of stable coronary artery disease The Task Force on the management of stable coronary artery disease. Eur. Heart J..

[2] Fihn SD (2014). 2014 ACC/AHA/AATS/PCNA/SCAI/STS focused update of the Guideline for the diagnosis and management of patients with stable ischemic heart disease. A Report of the American College of Cardiology/American Heart Association Task Force on Practice Guidelines, an. Circulation.

[3] Toth G (2014). Evolving concepts of angiogram: Fractional flow reserve discordances in 4000 coronary stenoses. Eur. Heart J..

[4] Van Nunen LX (2015). Fractional flow reserve versus angiography for guidance of PCI in patients with multivessel coronary artery disease (FAME): 5-year follow-up of a randomised controlled trial. Lancet.

[5] Pijls NHJ (2007). Percutaneous coronary intervention of functionally nonsignificant stenosis. J. Am. Coll. Cardiol..

[6] Pijls NHJ (2010). Fractional flow reserve versus angiography for guiding percutaneous coronary intervention in patients with multivessel coronary artery disease: 2-year follow-up of the FAME (fractional flow reserve versus angiography for multivessel evaluation) study. J. Am. Coll. Cardiol..

[7] Dattilo PB, Prasad A, Honeycutt E, Wang TY, Messenger JC (2012). Contemporary patterns of fractional flow reserve and intravascular ultrasound use among patients undergoing percutaneous coronary intervention in the United States insights from the National Cardiovascular Data Registry. J. Am. Coll. Cardiol..

[8] Westra J (2018). Diagnostic performance of in-procedure angiography-derived quantitative flow reserve compared to pressure-derived fractional flow reserve: The FAVOR II Europe-Japan study. J. Am. Heart Assoc..

[9] Xu B (2017). Diagnostic accuracy of angiography-based quantitative flow ratio measurements for online assessment of coronary stenosis. J. Am. Coll. Cardiol..

[10] Westra J (2018). Evaluation of coronary artery stenosis by quantitative flow ratio during invasive coronary angiography: The WIFI II study (wire-free functional imaging II). Circ. Cardiovasc. Imaging.

[11] Tu S (2016). Diagnostic accuracy of fast computational approaches to derive fractional flow reserve from diagnostic coronary angiography. JACC Cardiovasc. Interv..

[12] Collet C (2018). Diagnostic performance of angiography-derived fractional flow reserve: A systematic review and Bayesian meta-analysis. Eur. Heart J..

[13] Yazaki K (2017). Applicability of 3-dimensional quantitative coronary angiography-derived computed fractional flow reserve for intermediate coronary stenosis. Circ. J..

[14] Smit JM (2018). Referral of patients for fractional flow reserve using quantitative flow ratio. Eur. Hear. J. Cardiovasc. Imaging.

[15] Knuuti J (2019). 2019 ESC Guidelines for the diagnosis and management of chronic coronary syndromes. Eur. Heart J..

[16] Tu S (2014). Fractional flow reserve calculation from 3-dimensional quantitative coronary angiography and TIMI frame count. JACC Cardiovasc. Interv..

[17] Matsumura M (2017). Accuracy of fractional flow reserve measurements in clinical practice: Observations from a core laboratory analysis. JACC Cardiovasc. Interv..

[18] Park S-J (2012). Visual-functional mismatch between coronary angiography and fractional flow reserve. JACC Cardiovasc. Interv..

